# Supplementary material for: Reconciling Mining with the Conservation of Cave Biodiversity: A Quantitative Baseline to Help Establish Conservation Priorities
Source: PLoS One. 2016 Dec 20;11(12):e0168348. doi: 10.1371/journal.pone.0168348 (PMC5173368; doi:10.1371/journal.pone.0168348)
Supplement: S1 Dataset — (ZIP) [file pone.0168348.s002.zip › Taxa/Serra Norte/SN_2013/N4WS/Lista N4WS-47.pdf]

CAVIDADE N4WS-0047

Classe	Ordem	Fam/Outros	Gên/Outros	Espécie	S-U	U-S	Zon.
Annelida	Oligochaeta			sp.		4	P
Arachnida	Acari	(Oribatida)		sp.1	X	X	P
Arachnida	Acari	(Parasitengonina)		sp.1		X	P
Arachnida	Acari	(Parasitengonina)		sp.3		X	P
Arachnida	Acari	Ixodidae	*Amblyomma*	sp.1	X		P
Arachnida	Acari	Rhagidiidae		sp.1		X	P
Arachnida	Amblypygi	Phrynidae	*Heterophrynus*	*longicornis*	1	2	P
Arachnida	Araneae	Araneidae		jovem	X		E
Arachnida	Araneae	Caponiidae		jovem		X	P
Arachnida	Araneae	Ctenidae		jovem		1	E
Arachnida	Araneae	Mysmenidae		jovem	X		E/P
Arachnida	Araneae	Ochyroceratidae	*Speocera*	sp.1		X	P
Arachnida	Araneae	Pholcidae	*Mesabolivar*	*aurantiacus*	X		E
Arachnida	Araneae	Pholcidae	*Mesabolivar*	*eberhard*	X		P
Arachnida	Araneae	Pholcidae		jovem		X	P
Arachnida	Araneae	Salticidae		jovem	X		E
Arachnida	Araneae	Theridiidae		sp.1	X		P
Arachnida	Araneae	Theridiosomatidae	*Plato*	sp.1	X	X	P
Arachnida	Araneae	Uloboridae	*Uloborus*	sp.1	X	X	P
Arachnida	Opiliones	Cosmetidae	*Roquettea*	*singularis*	X		E
Arachnida	Palpigradi	Eukoeneniidae	*Eukoenenia*	sp.1		X	P
Arachnida	Pseudoscorpiones	Chernetidae	*Spelaeochernes*	sp.1	X	X	P
Arachnida	Pseudoscorpiones	Chthoniidae	*Pseudochthonius*	sp.1		X	P
Diplopoda	Spirostreptida	Pseudonannolenidae	*Pseudonannolene*	sp.2		1	P
Entognatha	Collembola	Isotomidae		sp.1		X	P
Entognatha	Collembola	Paronellidae		sp.1	X	X	E/P
Entognatha	Collembola	Paronellidae		sp.4		X	P
Entognatha	Diplura	Campodeidae		sp.1	X	X	E/P
Insecta	Blattodea			jovem	1		P
Insecta	Coleoptera	Carabidae		jovem		X	P
Insecta	Coleoptera	Scarabaeidae		jovem	X		P
Insecta	Coleoptera	Scydmaenidae		sp.8		X	P
Insecta	Coleoptera	Tenebrionidae		jovem		X	P
Insecta	Diptera	Cecidomyiidae	Cecidomyiinae	sp.	X		E/P
Insecta	Diptera	Ceratopogonidae		sp.	X		P
Insecta	Diptera	Chironomidae		sp.	X		E
Insecta	Diptera	Psychodidae	*Micropygomyia*	*pilosa*	X		E
Insecta	Diptera	Psychodidae	*Psychodopygus*	Série *chagasi*		X	P
Insecta	Diptera	Psychodidae	*Sciopemyia*	*sordellii*	X		P
Insecta	Diptera	Sciaridae	*Bradysia*	sp.		X	P
Insecta	Diptera	Tipulidae	Tipulinae	sp.	X		E/P
Insecta	Heteroptera	Lygaeidae	Rhyparochrominae	sp.1		X	E
Insecta	Heteroptera	Reduviidae	Reduviinae	jovem	1		E
Insecta	Homoptera	Cixiidae		sp.3	X	X	P
Insecta	Hymenoptera	Formicidae	*Acromyrmex*	sp.1	X		E
Insecta	Hymenoptera	Formicidae	*Camponotus*	sp.1	X		P
Insecta	Hymenoptera	Formicidae	*Dolichoderus*	aff. *bispinosus*		X	E/P
Insecta	Hymenoptera	Formicidae	*Gnamptogenys*	*striatula*	X		E/P
Insecta	Hymenoptera	Formicidae	*Nylanderia*	sp.2	X		E

Insecta	Hymenoptera	Formicidae	*Solenopsis*	sp.2		X	P
Insecta	Hymenoptera	Formicidae	*Strumigenys*	sp.1		X	P
Insecta	Hymenoptera	Formicidae	*Wasmannia*	sp.1	X	X	E/P
Insecta	Isoptera	Termitidae	*Armitermes*	sp.	X		E
Insecta	Isoptera	Termitidae	*Nasutitermes*	sp.	X		E/P
Insecta	Lepidoptera	Noctuoidea		sp.2	2		P
Insecta	Lepidoptera	Noctuoidea		sp.12		X	P
Insecta	Orthoptera	Phalangopsidae	*Paraclodes*	sp.	11	5	E/P
Insecta	Orthoptera	Phalangopsidae	*Phalangopsis*	sp.	5	10	P
Insecta	Psocoptera	Psocomorpha		jovem		X	E/P
Amphibia	Anura	Strabomantidae	*Pristimantis*	cf. *fenestratus*	3		P
Mammalia	Chiroptera	Emballonuridae	*Peropteryx*	sp.	2	1	P
